# An improved ontological representation of dendritic cells as a paradigm for all cell types

**DOI:** 10.1186/1471-2105-10-70

**Published:** 2009-02-25

**Authors:** Anna Maria Masci, Cecilia N Arighi, Alexander D Diehl, Anne E Lieberman, Chris Mungall, Richard H Scheuermann, Barry Smith, Lindsay G Cowell

**Affiliations:** 1 Department of Biostatistics and Bioinformatics, Duke University Medical Center, Durham, NC, USA; 2 Department of Cellular and Molecular Biology and Pathology, University of Naples, Naples, Italy; 3 Laboratory of Immunobiology of Cardiovascular Diseases, Department of Medical Science and Rehabilitation, IRCCS San Raffaele Pisana, Roma, Italy; 4 Protein Information Resource, Georgetown University Medical Center, Washington, DC, USA; 5 Mouse Genome Informatics, The Jackson Laboratory, Bar Harbor, ME, USA; 6 Lawrence Berkeley National Laboratory, Berkeley CA, USA; 7 Department of Pathology, Division of Biomedical Informatics, UT Southwestern Medical Center, Dallas, TX, USA; 8 Department of Philosophy and Center of Excellence in Bioinformatics and Life Sciences, University at Buffalo, Buffalo, NY, USA

## Abstract

**Background:**

Recent increases in the volume and diversity of life science data and information and an increasing emphasis on data sharing and interoperability have resulted in the creation of a large number of biological ontologies, including the Cell Ontology (CL), designed to provide a standardized representation of cell types for data annotation. Ontologies have been shown to have significant benefits for computational analyses of large data sets and for automated reasoning applications, leading to organized attempts to improve the structure and formal rigor of ontologies to better support computation. Currently, the CL employs multiple *is_a *relations, defining cell types in terms of histological, functional, and lineage properties, and the majority of definitions are written with sufficient generality to hold across multiple species. This approach limits the CL's utility for computation and for cross-species data integration.

**Results:**

To enhance the CL's utility for computational analyses, we developed a method for the ontological representation of cells and applied this method to develop a dendritic cell ontology (DC-CL). DC-CL subtypes are delineated on the basis of surface protein expression, systematically including both species-general and species-specific types and optimizing DC-CL for the analysis of flow cytometry data. We avoid multiple uses of *is_a *by linking DC-CL terms to terms in other ontologies via additional, formally defined relations such as *has_function*.

**Conclusion:**

This approach brings benefits in the form of increased accuracy, support for reasoning, and interoperability with other ontology resources. Accordingly, we propose our method as a general strategy for the ontological representation of cells. DC-CL is available from .

## Background

In the last decade, technological developments have resulted in tremendous increases in the volumes and diversity of the data and information that must be processed in the course of biomedical and clinical research and practice, and researchers are under ever greater pressure from funding agencies to share data and to take steps to ensure that data resources are interoperable. The use of ontologies to annotate data has proven successful in supporting these goals and in providing new possibilities for the automated processing of data and information [1-5]. This success has, in turn, resulted in the creation of a large number of ontologies, now made available through the Open Biological Ontologies repository [6] and through the BioPortal of the National Center for Biomedical Ontology [7].

More recently, ontologies have been shown to have significant benefits for the analysis of data resulting from high-throughput technologies [8] and for automated reasoning applications [9-12], which has led to organized attempts to improve the structure and formal rigor of ontologies in ways that will better support computational analysis and reasoning [13].

To help meet the data annotation needs of model organism researchers, Bard and colleagues developed an ontology of cell types [14] that has been widely used for the annotation of data in genome and other biological databases, including biorepository data. To enhance the CL's utility for computational analyses, we developed a systematic approach for the ontological representation of cells as described below, adhering to the principles outlined in [13].

The Cell Ontology (CL) currently contains representations of some 863 cell types, covering cell types from the major model organisms, including prokaryotic, fungal, animal, and plant cell types. This broad scope is accomplished primarily through the use of terms and definitions that can be applied to cell types from multiple types of organisms.

Cell types in CL are classified on the basis of a plurality of structural, histological, functional, and lineage properties. While some terms are provided with natural language definitions, most of the information in CL is conveyed through hierarchical classifications of the cell types along the following multiple axes of classification:

   *cell by organism*

   *cell by histology*

   *cell by function*

   *cell by lineage*

   *cell by nuclear number*

   *cell by ploidy*.

Each cell type within the CL is related to at least one supertype via the *is_a *(subtype) relation, but most CL cell types have multiple supertypes, and are thus classified on multiple axes, a phenonmenon referred to as '*is_a *overloading' [15]. CL is currently undergoing review, addressing specifically the problems that arise from the use of multiple modes of classification.

**Figure 1:**
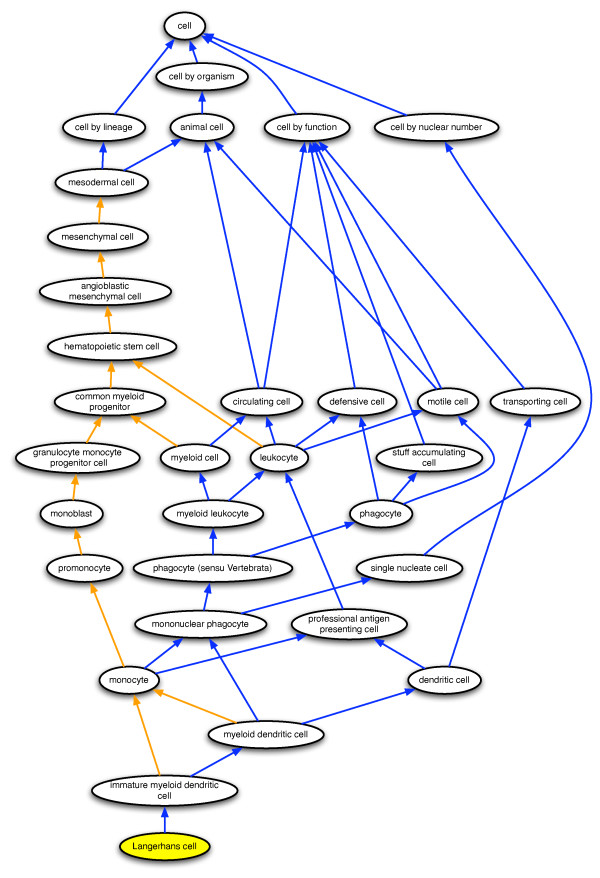
**The representation of Langerhans cells in the Cell Ontology**. A portion of the Cell Ontology is shown with ovals corresponding to cell types defined in the ontology and arrows corresponding to relations between those cell types. Langerhans cell is represented by a yellow oval; blue arrows correspond to *is_a *relations, and orange arrows correspond to *develops_from *relations. Only a subset of Langerhans cell parent types are included in the figure.

An example of the CL's use of multiple hierarchies is classification of *Langerhans cell *within the *cell by organism *and *cell by nuclear number *hierarchies as a subtype of *animal cell *and *single nucleate cell*, respectively (Figure 1). *Langerhans cell *is further classified within the functional hierarchy (Figure 1) as a subtype of:

   *transporting cell*

   *professional antigen presenting cell*

   *circulating cell*

   *motile cell*

   *defensive cell*

   *phagocyte (sensu Vertebrata)*.

Following a path along both *is_a *and *develops_from *relations – the latter is the relation "used to code developmental lineage relationships" – reveals that Langerhans cells are of mesodermal and hematopoietic lineage (Figure 1) [14]. *is_a *and *develops_from *are the only relations used in the current version of the CL.

To enhance the CL's utility for computational analyses, we developed a systematic approach for the ontological representation of cells that:

i) separates classification via the *is_a *relation from the assertion of structural, functional, and lineage properties by using formally defined, property-specific relations, such as *has_function*

ii) systematically includes both species-neutral and species-specific representations of cell types

iii) defines cell types on the basis of specific combinations of surface proteins used for identification of the cells via flow cytometry.

We have applied our method to develop an extension of the CL, DC-CL, for dendritic cells (DC), focusing on the DC types observed in mice and humans. The approach we propose increases the amount and accuracy of information contained in DC-CL, enhances its support for cross-species data integration, and optimizes it for the analysis of flow cytometry data.

The CL currently contains representations of six DC types:

   *dendritic cell*

   *plasmacytoid dendritic cell*

   *myeloid dendritic cell*

   *immature myeloid dendritic cell*

   *mature myeloid dendritic cell*

   *Langerhans cell*

which are defined on the basis of structural, functional, and lineage characteristics. The definitions are formulated in a highly general way to ensure broad applicability, but unfortunately at the cost of a precise specification of cell types. For example, *plasmacytoid dendritic cell *is defined as:

A dendritic cell type of distinct morphology, localization, and surface marker expression from other dendritic cell types and associated with early stage immune responses, particularly the release of physiologically abundant amounts of type 1 interferon in response to infection.

In the immunological literature and research community, the term 'dendritic cell' does not refer to a single, clearly distinguishable cell type; rather it refers to cells from a variety of sub-populations that have different morphologies, are distributed across different microenvironments within the body, express different microbial receptors and surface molecules, and different cytokines [16-18]. Cells in the various subpopulations are referred to using a common term because they are optimized to play a particular role in an immune response, that of priming an immune response by stimulating naïve T cells in the T cell zones of secondary lymphoid tissue to proliferate and execute their effector activities, but the cells in the various sub-populations are equipped to detect different pathogens and modulate distinct classes of immune responses. The structural, functional, and lineage similarities and differences between these sub-populations are not yet well understood, however, and their study is challenging because they are sensitive to changes in the cells' microenvironment. The subpopulations are typically characterized using a combination of variables, including flow cytometry or immunohistochemistry markers, function, and anatomical location, but the issue of how to define distinct DC subtypes is still an area of active debate within the immunology community.

We chose DCs as our case study in order to standardize the current terminology and definitions for DC subtypes and provide a common point of reference from which to maintain a common representation of DCs as knowledge about their subtypes evolves. DC-CL employs a flexible framework, which can be amended where necessary as knowledge advances. DC-CL can be used as a reference for the design and description of experiments, the interpretation of experimental results, and the integration of data from different sources, thereby facilitating progress towards a detailed, shared understanding of DC subtypes and/or their roles in immunity and tolerance.

## Methods

To develop a general method for the ontological representation of cell types, we first identified five families of cellular properties that can hold for a given cell type across the various microenvironments in which the cell type can be found. We identified the following five such families of properties:

i) structural components, such as granules in the cytoplasm or molecules on the cell surface,

ii) functions cells of the given type perform,

iii) dispositions, such as the tendency to participate in certain types of processes,

iv) anatomical locations in which the cells are found, and

v) lineage relationships.

**Figure 2:**
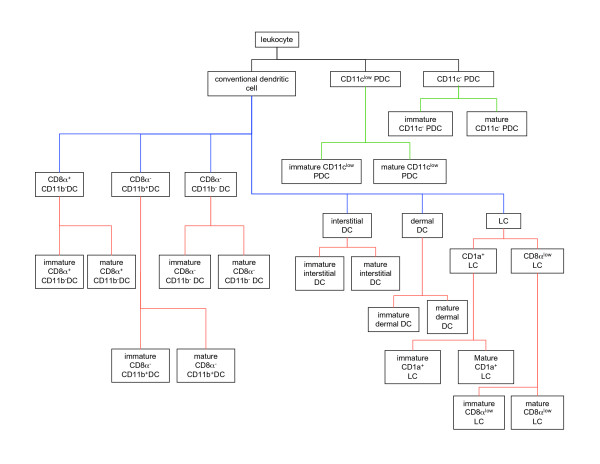
**The representation of dendritic cell types in the Dendritic Cell Ontology (DC-CL)**. Rectangles correspond to the terms for dendritic cell types represented in DC-CL, and the lines connecting the rectangles correspond to the *is_a *relations between these cell types. Black lines connect the highest-level terms in DC-CL to the Cell Ontology term *leukocyte*. Blue lines connect the DC-CL term *conventional dendritic cell *to the terms for its subtypes, while red lines connect these terms to the respective subtype terms. The green lines connect the terms *CD11c*^- ^*plasmacytoid dendritic cell *and *CD11c*^*low *^*plasmacytoid dendritic cell *to the terms for their respective subtypes. Abbreviations used in the figure are: DC, dendritic cell; PDC, plasmacytoid dendritic cell; and LC, Langerhans Cell.

Among these properties, we emphasize structural components, as these are most often used to identify cell types for study and because their use for classification facilitates maintenance of a single-hierarchy classification of DC types (Figure 2). More precisely, we classify DCs by surface protein expression. For other types of cells, other structural components may be used, as for example in the classification of eosinophils, basophils, and neutrophils, which can be classified on the basis of types of cytoplasmic granules.

**Figure 3:**
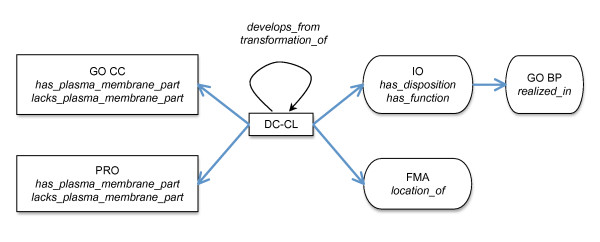
**The ontologies and relations referred to in the Dendritic Cell Ontology (DC-CL)**. The rectangles and ovals represent ontologies, and the arrows represent relations joining terms in the ontologies. Abbreviations for the ontology names are shown in normal font, and the relations used to link DC-CL to each ontology are shown in italics. The black arrow indicates relations used to join DC-CL terms to other DC-CL terms, while the blue arrows indicate trans-ontological relations. Ontologies and relations shown in rectangles are used to define DC-CL types, while the ontologies and relations shown in ovals are used to make non-classificatory assertions about DC-CL types. Abbreviations used in the figure are: GO CC, Gene Ontology Cellular Component Ontology; PRO, Protein Ontology; IO, Immunology Ontology; FMA, Foundational Model of Anatomy; and GO BP, Gene Ontology Biological Process Ontology.

We next identified a set of ontology development principles designed to maximize both an ontology's utility for computational analysis and reasoning and its interoperability with existing resources. The basis of this approach is use of relations from the OBO Foundry Relation Ontology (RO) [19] to link terms both within DC-CL and also from DC-CL to other OBO Foundry ontologies (Figure 3), as described in [13,20]. Relations in the RO are formally defined to support automated reasoning [19]. In addition, in constructing DC-CL we employed accepted principles of ontology development outlined by the OBO Foundry [13], including the use of genus-differentia definitions [21].

We use RO relations wherever possible, and define new relations where needed, following the approach used in RO. Relations in RO are defined in accordance with the distinction between types and instances [19], corresponding to the standard Description Logic distinction between A-boxes and T-boxes used within the OWL/Semantic Web community [22]. Types are *general*; they are the kinds of things that exist and are documented in scientific textbooks (e.g. human, red blood cell); instances are particular; they are the specific examples upon which experiments are performed (e.g. J. Craig Venter, the many red blood cells in my body). The relations between types represented in an ontology are defined in terms of relations between the corresponding instances of those types of the sort that can be observed in experiments. Thus, a type-level relation *R *will be defined in terms of the corresponding instance-level relation **R **as follows [where *italics *indicates type-level relations, **bold **indicates instance-level relations, type-level variables are denoted by upper case (X, Y, Z, ...), and instance-level variables are denoted by lower case italics (*x, y, z*, ...)]:

X *stands_in_R_to *Y = _def _for every instance *x *of type X, there exists at least one instance *y *of type Y, such that *x ***stands_in_R_to ***y*.

We use *R*(X, Y) and X*R*Y to abbreviate: all Xs stand in **R **to some Y. Thus, *has_part*(human, brain) abbreviates: every instance of *human *has some instance-level part which is an instance of *brain*.

Defining type-level relations in terms of *all *instances of X ensures that assertions of relations between types hold universally, i.e. X*R*Y will hold only if *all *instances of X stand in **R **to *some *instance of Y. This universality in turn ensures the possibility of transitive reasoning by ensuring that relations transitive at the instance level are transitive at the type level. Thus, if **R **is transitive and *R *is defined in terms of **R **and *all *instances of X, then, if X*R*Y holds at time t and Y*R*Z holds at t, X*R*Z also holds at t.

### Structural Components

#### Defining cell types in terms of surface proteins

Cell types in DC-CL are defined in terms of the proteins and protein complexes expressed on the cell surfaces of the corresponding instances, consistent with the characterization of DC populations using surface protein expression [23]. The definitions of such types thus involve terms representing proteins in the Protein Ontology (PRO) [24] and protein complexes in the Gene Ontology Cellular Component Ontology (GO CC) [25]. The reference to proteins and protein complexes enables us to assert the specific presence or absence of defining molecules on cells of a given type. To make these assertions, we define the relations *has_plasma_membrane_part *and *lacks_plasma_membrane_part *in terms of the RO instance-level relations **has_part **and **instance_of **and the GO CC term *plasma membrane*:

We first define the instance-level relation

   *c ***has_plasma_membrane_part ***p ***at ***t *= _def _there exists some *m*, such that

      *m ***instance_of ***plasma membrane *at *t*

      *c ***has_part ***m ***at ***t*

      *m ***has_part ***p ***at ***t*.

We can then define:

   C *has_plasma_membrane_part *P = _def _for all *c *and all times *t*, if *c ***instance_of **C at

   *t*, then there exists some *p *such that *p ***instance_of **P and *c*

   **has_plasma_membrane_part ***p *at *t*

   C *lacks_plasma_membrane_part *P = _def _for all *c *and all times *t*, if *c ***instance_of **C at

   *t*, then there is no *p *such that *p ***instance_of **P and *c*

   **has_plasma_membrane_part ***p *at *t*

C *has_part *P is implied by C *has_plasma_membrane_part *P because both the **has_part **and *has_part *relations are transitive. Neither **has_plasma_membrane_part **nor *has_plasma_membrane_part *is transitive, however.

Note that there is an important distinction between the two expressions:

   C *lacks_plasma_membrane_part *P

   It is not the case that C *has_plasma_membrane_part *P.

The former asserts that there is *no *instance of the type C that *has *an instance of the type P as a part of its plasma membrane; the latter asserts only that there is *at least one *instance of the type C that has no instance of the type P as part of its plasma membrane.

Using the above-defined relations, definitions for DC-CL types take the form:

   CD11c^- ^plasmacytoid dendritic cell *is_a *leukocyte_CL _that

      *has_plasma_membrane_part *CD45RA_PRO _and

      *has_plasma_membrane_part *CD123_PRO _and

      *has_plasma_membrane_part *CD303_PRO _and

      *has_plasma_membrane_part *ILT7_PRO _and

      *lacks_plasma_membrane_part *CD11c_PRO _and

      *lacks_plasma_membrane_part *CD3_PRO _and

      *lacks_plasma_membrane_part *CD19_PRO _and

      *lacks_plasma_membrane_part *CD34_PRO _and

      *lacks_plasma_membrane_part *CD56_PRO_

where the subscripts indicate the ontology source for each term. DC-CL, PRO, and GO CC are thereby linked through the assertion of these trans-ontological relations.

Because no canonical CD11c^- ^plasmacytoid DC expresses CD11c on its cell surface, our definition correspondingly includes the assertion: *lacks_plasma_membrane_part *CD11c. On the other hand, because some CD11c^- ^plasmacytoid DCs will express CCR7 on their surface while others will not, a *lacks_plasma_membrane_part *CCR7 assertion is not valid for DCs of this type, and neither is a *has_plasma_membrane_part *CCR7 assertion. The negation of the *has_plasma_membrane_part *CCR7 assertion is valid in this case, however, and we are exploring the issue of whether assertions of this sort could be useful for the construction of defined classes, collections of cells that do not constitute distinct types yet are identifiable as a group as a consequence of common but not defining characteristics [26]. For example, two defined classes of plasmacytoid DCs might be distinguished, those that do and those that do not express CCR7 on their respective membranes.

#### Defining cell types by differences in protein expression levels

For some cell types, including DCs, specification of the *level *of protein expression is necessary for defining the cell type [23,27,28]. Cell types are said to express low or high levels of a protein, the salient amount being relative to the distribution of expression levels among a defined reference population of cells. The relevant reference population of cells is selected on the basis of the cells' possession of certain physical characteristics, such as a particular size and shape or pattern of surface marker expression. In flow cytometry experiments, measurements of forward angle and orthogonal (side) light scatter are used as surrogate measures of cell size and organelle complexity. The relevant population of cells can be defined by forward and side scatter parameters alone, or by using these parameters in conjunction with the expression level of surface proteins additional to the protein of interest.

In the analysis of flow cytometry data, the populations of cells used to generate the reference distribution of expression levels are defined specifically for the context of each experiment. To define cell types ontologically, we select a fixed cell type and refer to the distribution of expression levels among instances of the type in our definition of relations and cell types. For each cell type definition, we select this reference cell type, *C*_*R*_, such that the cell type definition referring to *C*_*R *_holds even when instances of the cell type are identified in a particular experiment by protein expression levels relative to a different (i.e. more restricted) population of cells. For example, CD11c^low ^plasmacytoid DCs express low levels of CD11c relative to leukocytes and are defined this way in DC-CL. In flow cytometry experiments, however, such cells are frequently analyzed in reference to a restricted population of leukocytes from which T cells and NK T cells have been removed.

To include levels of expression in the definition of cell types in DC-CL, we define the two tertiary relations *has_high_amount*(X, Y, *C*_*R*_) and *has_low_amount*(X, Y, *C*_*R*_) that allow us to assert, for example, that entities of type X contain a high amount of Y, where 'high' is defined relative to the distribution of expression levels of Y among entities of type *C*_*R*_. These relations will be used to make a series of assertions about the amount of protein Y expressed by cell type X relative to the amount of protein Y expressed by a reference cell type *C*_*R*_. Such assertions are needed for example to distinguish conventional DCs (CD11c high), CD11c low plasmacytoid DCs, and CD11c negative plasmatcytoid DCs.

To define these relations, we first define the functional operators *geometric_mean_of *and *number_of*. The first is defined in the usual way, as a function from a set of numbers to their geometric mean [29]. *number_of*(*x*, Y, **R**, *t*) is defined as the number of instances of type Y that are related to *x*, an instance of X, by the relation **R **at time *t*. Thus for example where **R **is the relation **has_ part**, this results in:

   *number_of*(*x*, Y, **has_ part**, *t*) = _def _the number of instances *y *of Y at time *t *such that *x ***has_part ***y *at *t*

This defines a functional mapping, which yields, for a given cell instance *x *and time *t*, the number of entities of a given type Y (for example: number of instances of a certain type of molecule) which are part of that cell at that time.

Using these functional operators, we can define *has_high_amount *as follows:

   *has_high_amount *(X, Y, Z) = _def _for all *x *and all times *t*, if *x ***instance_of **X at *t*, then there exist some *y*, *y*' and *z*, such that:

      *y ***instance_of **Y at *t*

      *y*' **instance_of **Y at *t*

      *z ***instance_of **Z at *t*

      *x ***has_part ***y *at *t*

      *z ***has_part ***y*' at *t*,

      *number_of*(*x*, Y, **has_part**, *t*)

         > *geometric_mean_of*({*number_of*(*z*, Y, **has_part**, *t*) : *z ***instance_of **Z at t})

where Z is a reference cell type, as defined above.

Thus, *has_high_amount*(X, Y, Z) asserts that, for each instance *x *of X and for all times at which *x *is an instance of X, there exist instances *y *of Y that are part of *x*, and that the number of such instances y is greater than the geometric mean number of instances that are part of an instance of Z, the reference cell type. *has_low_amount*(X, Y, Z) is similarly defined, substituting the less than (<) relation for the greater than (>) relation. In the context of DC-CL, X and Z are cell types, *y *is a protein or protein complex, and Y is the corresponding molecule type.

To specify relative amounts of surface expression, we define

   *has_high_plasma_membrane_amount*(X, Y, Z)

   *has_low_plasma_membrane_amount*(X, Y, Z)

as above, but using **has_plasma_membrane_part **in place of **has_part **in the specification of the relevant *number_of *operators.

In DC-CL, we use *leukocyte *as common reference cell type, so that the expression levels of a particular molecule on DC subtypes are relative to the distribution of expression levels of that molecule on leukocytes. For convenience, we define *has_high_plasma_membrane_amount_relative_to_leukocyte*(X, Y) as *has_high_plasma_membrane_amount*(X, Y, *leukocyte*), and similarly X *has_low_plasma_membrane_amount_relative_to_leukocyte *Y.

As an example of a DC-CL definition using the

*has_high_plasma_membrane_amount_relative_to_leukocyte *relation, consider the definition of dermal DCs as having as part CD11b and CD205 (i.e. being CD11b positive and CD205 positive; CD11b^+ ^CD205^+^), having high amounts of CD11c (CD11c^high^), and lacking CD8α (CD8α^-^):

   dermal dendritic cell_DC-CL _*is_a *leukocyte_CL _that

      *has_plasma_membrane_part *CD11b_PRO _and

      *has_plasma_membrane_part *CD205_PRO _and

      *has_high_plasma_membrane_amount_relative_to_leukocyte *CD11c_*PRO *_and

      *lacks_plasma_membrane_part *CD8α homodimer_GO CC_

To assess whether the geometric mean represents an appropriate threshold to distinguish high and low expressing cells, we analyzed histograms of fluorescence intensities for CD11c, CD11b, CD45R, CD80, CD86, and MHC II under the assumption that the number of molecules of the relevant type on the cell surface is correlated with the fluorescence intensity after staining for the molecule of interest. Spleens were removed from untreated mice, erythrocytes were lysed, and the cells were incubated with the appropriate fluorescence-labeled antibody or isotype control, as described in [30]. Histograms for each molecule type were generated after gating on appropriate forward and side scatter values to exclude dead cells and gating on fluorescence intensity to exclude cells that do not express the molecule of interest. For each histogram, the geometric mean of fluorescence intensities was computed and evaluated as a threshold for classifying high versus low expressing cells (data not shown). We found that the geometric mean computed on the fluorescence intensities of positive cells provides good discrimination between high and low expressing cells. While a formal analysis of the utility of the geometric mean of fluorescence intensities for the classification of cells remains to be carried out and is beyond the scope of this paper, this initial analysis provides support for the use of the geometric mean in the definition of the relations *has_high_amount *and *has_low_amount*.

#### Species specificity of DC-CL cell types

To best facilitate cross-species data interoperability, ontologies need to provide both species-neutral and species-specific terms. Species-neutral terms allow for commonalities between species to be identified. Such commonalities can be used for the generation of hypotheses not only about human biology from experimental results observed in model organisms but also about basic principles, such as those underlying cell biology or mammalian biology. Species-specific terms are equally important, however, as they allow for the more accurate and precise representation of information and data pertaining to organisms of different species and thus enable us to capture more precisely the differences between such organisms.

The approach we outline for defining cell types in DC-CL results in the systematic inclusion of both species-neutral and species-specific terms and allows prospectively for the systematic capture of orthology relationships between species-specific types, where they exist. Because we define cell types by linking to species-neutral terms in PRO and GO CC, cells expressing the same combination of surface proteins are instances of the same DC-CL type, regardless of their species of origin. For example, cells that are CD11c^+ ^and CD19^- ^CD3^- ^C34^- ^CD56^- ^are referred to as conventional dendritic cells in both mice and humans, and instances of such cells in mice and in humans are instances of the DC-CL type *conventional dendritic cell*. Species-specific terms arise not because we use species of origin as a defining characteristic, but rather because the DC types identified in mice and humans express different combinations of surface proteins. For example, the cells referred to as plasmacytoid dendritic cells in mice are marked by expression of CD45R, GR1, and CD11c and the absence of CD11b, while in humans, plasmacytoid dendritic cells are marked by expression of CD45RA, CD123, CD303, and ILT7. Thus, in DC-CL, we have CD11c^+ ^and CD11c^- ^plasmacytoid dendritic cells, corresponding to those observed in mice and humans, respectively.

PRO includes multiple levels of classification for proteins, including a gene product level, a sequence variant level (e.g. for genetic differences), a protein isoform level (e.g. for splice variants), and a post-translational modification level [24,31]. PRO is species-neutral in the sense that, proteins from different species that are the products of orthologous genes are instances of the same PRO gene product level type, and proteins from different species representing equivalent forms (e.g. CD45RA in human and mouse) are instances of the same sequence variant level or protein isoform level types. Likewise, protein forms from different species with equivalent post-translational modifications are instances of the same post-translational modification level type. The protein complex types represented in GO CC are similarly species-neutral. Although the curation of PRO has thus far focused on inclusion of types corresponding to the protein instances observed in mice and humans, future PRO curation efforts will ensure broader species inclusivity.

To avoid the unnecessary creation of overly specific subtypes, we define cell types in terms of PRO gene product level types, and recommend that PRO types at this level be used wherever possible. When necessary, however, representations of more specific protein types can be used. For example, the alternative splice forms CD45RA and CD45RO may be needed to define some T lymphocyte subtypes by linking to the corresponding PRO protein isoform level types [32].

When species-specific cell types are defined either through the use of species-specific combinations of PRO gene product level types or through the use of PRO types corresponding to species-specific amino acid sequences, the relationships between protein types in PRO provide the information needed to identify cell types expressing related proteins.

### Functions and Dispositions

The backbone *is_a *hierarchy that is used in formulating definitions of DC-CL terms is based on the types and levels of proteins expressed on the cell surface. DC-CL also includes, however, further relations that are used in assertions about the functions each of the cell types performs and about their dispositions to participate in processes of specific types. Assertions about functions are made by linking terms in DC-CL to terms referring to types of functions using the RO relation *has_function*. Similarly, assertions about dispositions are made using the RO relation *has_disposition*.

The terms for cellular functions and dispositions are linked to GO biological process (GO BP) terms using the RO relation *realized_in*, in axioms of the form:

   X *realized_in *Y_GO BP_

   X *realized_in *Y_GO BP_.

where Y is a process type from GO BP. For example:

   antigen processing activity_DC-CL _*realized_in *antigen processing and presentation_GO BP_

   disposition to cross-present antigen to CD8^+ ^T cells_DC-CL _*realized_in *antigen presentation, exogenous antigen via MHC class I_*GO BP*_.

Where the representation of functions and dispositions needed for DC-CL requires reference to a process for which we could find no corresponding GO BP term, we have submitted a term request to the Gene Ontology tracker [33].

### Locations

Assertions about the anatomical location of DC subtypes are made by relating DC subtype terms to terms in the Foundational Model of Anatomy [21], using assertions of the form:

   Y_FMA _*location_of *X_DC-CL_

which states that every instance of the anatomical structure type Y is the location of some instance of the DC type X. For example:

   lymph node_FMA _*location_of *mature CD8α^- ^CD11c^- ^dendritic cell_DC-CL_

While this is an ontological assertion about an anatomical entity rather than about a cell type, inclusion of assertions of this form allows the ontology to be queried for the anatomical locations in which the various DC types can be found. They also serve to link DC-CL to other ontologies within the OBO Foundry, and thus serve more general networking of information in a way that provides support for further types of reasoning.

### Lineage Relationships between Cell Types

Lineage relationships between cell types are captured using the *arises_from *and the *transformation_of *relations. The relations *arises_from*, *derives_from *and *transformation_of *are formally defined in the RO [19]. **derives_from**, the instance-level relation, is defined as the relation between "distinct material continuants when one succeeds the other across a temporal divide in such a way that at least a biologically significant portion of the matter of the earlier continuant is inherited by the latter" [19]. There are three types of **derives_from **relations, the continuation of an instance that loses a small portion of itself, fusion, such as the fusion of a sperm and an egg to form a zygote, and fission, such as the division of a cell to form two daughter cells. *derives_from*, the class-level relation, is the relation between classes C and C' when instances of C are connected to instances of C' by a series of **derives_from **relations [19]. *transformation_of *is defined as the relation between two classes, in which "one and the same continuant entity preserves its identity while instantiating distinct classes at distinct times" [19]. That is, a single instance is of type C at one time and of type C' at a later time, as in the transformation from child to adult. *arises_from *is the parent relation of *derives_from *and *transformation_of*.

In DC-CL, we use *transformation_of *as the relation between immature and mature cell types because we are asserting a one-to-one relationship between instances of the types. All other lineage relationships, such as that between hematopoietic stem cells and common lymphoid precursors, are asserted using the *arises_from *relation because the relationship between instances may not be one-to-one.

## Results

### DC-CL cell types

Terms for 29 DC types are defined in DC-CL, along with 12 precursor cell types, which are defined in order to assert lineage relationships. All DC types are subtypes of *conventional dendritic cell*, *CD11c*^*low *^*plasmacytoid dendritic cell*, or *CD11c*^- ^*plasmacytoid dendritic cell *(Table 1).

**Table 1:** The genus-differentia form of the definitions for the three most general types in DC-CL.

DC-CL Term	Genus	Differentia	
conventional dendritic cell	leukocyte_CL_	*has_high_plasma_membrane_amount*	CD11c_PRO_
		*lacks_plasma_membrane_part*	CD3_PRO_
		*lacks_plasma_membrane_part*	CD19_PRO_
		*lacks_plasma_membrane_part*	CD34_PRO_
		*lacks_plasma_membrane_part*	CD56_PRO_

CD11c^low^			
plasmacytoid dendritic cell	leukocyte_CL_	*has_low_plasma_membrane_amount*	CD11c_PRO_
		*has_plasma_membrane_part*	CD45R_PRO_
		*has_plasma_membrane_part*	GR1_PRO_
		*lacks_plasma_membrane_part*	CD11b_PRO_
		*lacks_plasma_membrane_part*	CD3_PRO_
		*lacks_plasma_membrane_part*	CD19_PRO_
		*lacks_plasma_membrane_part*	CD34_PRO_
		*lacks_plasma_membrane_part*	CD56_PRO_

CD11c^-^			
plasmacytoid dendritic cell	leukocyte_CL_	*has_plasma_membrane_part*	CD45RA_PRO_
		*has_plasma_membrane_part*	CD123_PRO_
		*has_plasma_membrane_part*	CD303_PRO_
		*has_plasma_membrane_part*	ILT7_PRO_
		*lacks_plasma_membrane_part*	CD11c_PRO_
		*lacks_plasma_membrane_part*	CD3_PRO_
		*lacks_plasma_membrane_part*	CD19_PRO_
		*lacks_plasma_membrane_part*	CD34_PRO_
		*lacks_plasma_membrane_part*	CD56_PRO_

The type *conventional dendritic cell *has 22 subtypes identified in the current version of DC-CL; *CD11c*^*low *^*plasmacytoid dendritic cell *and *CD11c*^- ^*plasmacytoid dendritic cell *each have 2 subtypes. The surface protein combinations that define these subtypes are shown in Tables 2 and 3, respectively; the placement of these types in the DC-CL hierarchy is shown in Figure 2.

**Table 2:** Surface protein combinations defining the subtypes of *conventional dendritic cell*.

	205	11b	4	8α	1a	206	209	36	14	207	324	80	83	86	MHC II
CD8α-CD11b- DC	+	-	-	-											
immature	+	-	-	-								L		L	L
CD8α-CD11b- DC															
mature	+	-	-	-								H	+	H	H
CD8α-CD11b- DC															

CD8α- CD11b+ DC	-	+	+	-											
immature	-	+	+	-								L		L	L
CD8α- CD11b+ DC															
mature	-	+	+	-								H	+	H	H
CD8α- CD11b+ DC															

CD8α + CD11b- DC	+	-	-	+											
immature	+	-	-	+								L		L	L
CD8α + CD11b- DC															
mature	+	-	-	+								H	+	H	H
CD8α + CD11b- DC															

interstitial DC		+			+	+	+								
immature		+			+	+	+					L		L	L
interstitial DC															
mature		+			+	+	+					H	+	H	H
interstitial DC															

Langerhans cell										+					
CD1a+ Langerhans cell					+					+	+				
immature CD1a+					+					+	+	L		L	L
Langerhans cell															
mature CD1a+					+					+	+	H	+	H	H
Langerhans cell															
CD8α^low ^Langerhans cell	H			L						+					
immature CD8α^low^	H			L						+		L		L	L
Langerhans cell															
mature CD8α^low^	H			L						+		H	+	H	H
Langerhans cell															

dermal DC	+	+		-											
immature dermal DC	+	+		-								L		L	L
mature dermal DC	+	+		-								H	+	H	H

Column headings show protein or protein complex names. Numbers correspond to CD antigens (e.g. CD205 is referred to as 205 in the table). Row labels show the terms for subtypes of *conventional dendritic cell *defined in DC-CL. Row labels not indented correspond to direct subtypes of *conventional dendritic cell*; indented row labels correspond to direct subtypes of the types referred to by the nearest, above row with less indent. + indicates that the *has_plasma_membrane_part *relation is use to join the cell type term to the protein or protein complex term; – indicates that the *lacks_plasma_membrane_part *relation is used; L indicates that the *has_low_plasma_membrane_amount *relation is used; and H indicates that the *has_high_plasma_membrane_amount *relation is used.

**Table 3:** Surface protein combinations defining the subtypes of *CD11c*^- ^*plasmacytoid dendritic cell *and *CD11c*^*low *^*plasmacytoid dendritic cell*.

	123	303	ILT7	45RA	45R	GR1	11c	11b	80	83	86	MHC II
immature CD11c^- ^plasmacytoid DC	+	+	+	+			-		-		L	L
mature CD11c^- ^plasmacytoid DC	+	+	+	+			-		H	+	H	H
immature CD11c^low ^plasmacytoid DC					+	+	L	-	L		L	
mature CD11c^low ^plasmacytoid DC					+	+	L	-	H	+	H	H

Other details are as in Table 2.

The precursor cell types are not DC subtypes, and are therefore not placed in the DC-CL hierarchy. We define them for completeness, however, in order to provide surface protein-based definitions for all cell types referred to in DC-CL assertions. The surface protein combinations that define the 12 precursor types are shown in Tables 4 and 5.

**Table 4:** Surface protein combinations used to define DC precursors in DC-CL.

	7	45RA	4	3, 19	34	123	10	71	133	33	2, 11b, 15, 56	16	11c	31	32	43	86
CD7^- ^lymphoid precursor	-	-	-	-	+												
CD7^+ ^lymphoid precursor	+	-	-	-	+												
granulocyte-monocyte precursor		+	-	-	+	+											
CD10^+ ^common lymphoid precursor	+	+	-	-	+		+										
CD71^+ ^common myeloid precursor	-	+	-	-	+		-	+									
CD133^+ ^hematopoietic stem cell				-	+			-	+	-	-	-					
CD43^+ ^monocyte										+			+	+	+	+	+

Other details as in Table 2.

**Table 5:** Surface protein combinations used to define DC precursors in DC-CL.

	117	16	135	M- CSFR	217	SCA1	2, 3, 4, 5, 8, 45R, GR1, NK1.1, Ter-119	32	34	90	115	11b
common dendritic precursor	+		+	+								
CD217+ common lymphoid precursor	+				+	+	-					
CD117+ common myeloid precursor	+	+			-	-	-	+	+			
SCA1^+ ^hematopoietic stem cell	+					+	-		-	-		
CD115^+ ^monocyte											+	+

Other details as in Table 2.

In addition to defining the DC types by the presence or absence of specific surface proteins, we include assertions about which of the Toll-like receptors (TLR) are expressed on each DC type. Future work on DC-CL will include incorporation of assertions about other molecules important to immune function, such as additional pattern recognition receptors and cytokine receptors.

### Functions and Dispositions

The DC types in DC-CL are related to functions (Table 6) and dispositions (Table 7) using the *has_function *and *has_disposition *relations. The six plasmacytoid dendritic cell types all share a common disposition to secrete type 1 interferon. Additional functions and dispositions for the subtypes of *conventional dendritic cell *and for the plasmacytoid dendritic cell subtypes are shown in Tables 8, 9 and 10.

**Table 6:** DC-CL function terms and their corresponding GO BP process terms.

**DC-CL Function Term**	**GO BP Process Term**
antigen capture activity	
antigen processing activity	dendritic cell antigen processing and presentation
antigen presentation activity	dendritic cell antigen processing and presentation
antigen transportation activity	
cytokine secretion activity	cytokine secretion

**Table 7:** DC-CL disposition terms and their corresponding GO-BP terms.

**DC-CL Disposition Term**	**GO BP Process Term**
disposition to circulate in the blood	
disposition to cross-present antigen to CD8+ T cells	antigen presentation, exogenous antigen via MHC class I
disposition to migrate to the lymph node	leukocyte migration
disposition to secrete anti-inflammatory cytokines	
disposition to secrete inflammatory cytokines	
disposition to secrete type 1 interferon	

**Table 8:** Functions of each conventional DC subtype.

	antigen processing activity	cytokine secretion activity	macro- pinocytosis activity	T cell antigen presentation activity	antigen transportation activity
CD8α-CD11b- DC		+		+	
immature	+	+	+	+	+
CD8α-CD11b- DC					
mature		+		+	
CD8α-CD11b- DC					

CD8α- CD11b+ DC		+		+	
immature	+	+	+	+	+
CD8α- CD11b+ DC					
mature		+		+	
CD8α- CD11b+ DC					

CD8α + CD11b- DC		+		+	
immature	+	+	+	+	+
CD8α + CD11b- DC					
mature		+		+	
CD8α + CD11b- DC					

interstitial DC		+		+	
immature	+	+	+	+	+
interstitial DC					
mature		+		+	
interstitial DC					

Langerhans cell		+		+	
CD1a+ Langerhans cell		+		+	
immature CD1a+	+	+	+	+	+
Langerhans cell					
mature CD1a+		+		+	
Langerhans cell					
CD8α^low ^Langerhans cell		+		+	
immature CD8α^low^	+	+	+	+	+
Langerhans cell					
mature CD8α^low^		+		+	
Langerhans cell					

dermal DC		+		+	
immature dermal DC	+	+	+	+	+
mature dermal DC		+		+	

+ indicates that the cell type referred to by the row label is related to the function indicated by the column heading using the *has_function *relation.

**Table 9:** Functions of each plasmacytoid DC subtype.

	antigen processing activity	cytokine secretion activity	macro- pinocytosis activity	T cell antigen presentation activity	antigen transportation activity
immature CD11c^-^	+	+	+	+	+
plasmacytoid DC					
mature CD11c^-^		+		+	
plasmacytoid DC					
immature CD11c^low^	+	+	+	+	+
plasmacytoid DC					
mature CD11c^low^		+		+	
plasmacytoid DC					

Other details as in Table 7.

**Table 10:** Dispositions of the conventional DC subtypes.

	disposition to circulate in the blood	disposition to cross-present antigen to CD8+ T cells	disposition to migrate to the lymph node	disposition to secrete anti- inflammatory cytokines	disposition to secrete inflammatory cytokines	disposition to secrete type 1 interferon
CD8α-CD11b- DC						
immature						
CD8α-CD11b-DC						
mature				+		
CD8α-CD11b-DC						

CD8α- CD11b+ DC				+		
immature				+		
CD8α- CD11b+DC						
mature				+		
CD8α- CD11b+DC						

CD8α + CD11b-DC		+			+	
immature		+			+	
CD8α + CD11b-DC						
mature		+			+	
CD8α + CD11b-DC						

interstitial DC						
immature			+			
interstitial DC						
mature						
interstitial DC						

Langerhans cell						
CD1a+						
Langerhans cell						
immature			+			
CD1a+						
Langerhans cell						
mature						
CD1a+						
Langerhans cell						
CD8α^low^			+		+	
Langerhans cell						
immature			+		+	
CD8α^low^						
Langerhans cell						
mature			+		+	
CD8α^low^						
Langerhans cell						

dermal DC						
immature			+			
dermal DC						
mature dermal DC						

+ indicates the cell type referred to by the row label is related to the disposition referred to by the column heading using the *has_disposition *relation.

Assertions in DC-CL are associated with reference to at least one journal article via PubMed ID.

## Discussion

We present here an ontology of DC types (DC-CL) and the method used to create the ontology. The motivation for developing DC-CL was two-fold: to provide a common point of reference for standardized terms and definitions for DC subtypes and to develop a method for representing cell types that is highly computable and builds on existing resources. DCs have a particularly complicated biology [16-18]; thus not only are efforts to develop standardized, comprehensive information resources needed, but DCs are a good model for testing a method for representing cells in an ontology.

We have developed DC-CL using a systematic approach for the ontological representation of cells that:

i) separates classification via the *is_a *relation from the assertion of structural, functional, and lineage properties by using formally defined, property-specific relations, such as *has_function*

ii) systematically includes both species-neutral and species-specific types

iii) defines cell types on the basis of specific combinations of surface proteins used for identification of the cells via flow cytometry.

The use of property-specific relations, such as *has_function*, to incorporate structural, functional, and lineage properties has many benefits. First, this approach eliminates many of the errors that frequently result from multiple uses of the *is_a *relation [34-36] in what has been called '*is_a *overloading' [15]. Second, the *is_a *relation can only be used between entities of the same ontological category (higher level types, such as those found in the Basic Formal Ontology described below), while specific relations can be used to relate cells to entities in other categories, such as functions (*has_function*), molecules (*has_part*), and processes (*participates_in*), that are represented within their own ontologies. DC-CL is formally connected to the hierarchical structure and relations of these ontologies, as well as the data annotated in their terms, thereby providing significant additional information and opportunities for data integration. The use of property-specific relations also allows us, without sacrificing expressive power, to maintain a policy of single inheritance (each representational unit in the ontology has maximally one single asserted *is_a *parent), which brings benefits such as clearer statement of definitions, easier and more reliable curation, ability to use more powerful reasoning tools, and the ability to have a unique measure of distance between any two terms on the same branch of an ontology. Finally, the use of property-specific relations enhances ontologies for computational analyses because each relation can be defined with its own inference properties.

The inclusion of species-specific cell types allows for the more specific annotation of data and for the incorporation within DC-CL of species-specific properties, many of which have important functional consequences. For example, the plasmacytoid DCs observed in humans (CD11c^-^) express Toll-like receptors (TLR) TLR7 and TLR9, while the plasmacytoid DCs observed in mice (CD11c^low^) express all mouse TLRs except for TLR3 and TLR4, with consequent differences in the types of pathogens human and mouse plasmacytoid DCs can detect [37]. We avoid use of species of origin as a basis of defining types, however, and only define types based on the presence or absence of specific surface proteins. Thus, the plasmacytoid DCs observed in humans are instances of the type *CD11c*^- ^*plasmacytoid dendritic cell*, while the plasmacytoid DCs observed in mice are instances of the type *CD11c*^*low *^*plasmacytoid dendritic cell*, where the two types are defined by the patterns of surface protein expression given in the above definitions. Plasmacytoid DCs observed in a third species to have either pattern of surface protein expression would be instances of the corresponding type. In addition, we only include assertions about the cell types that hold across all species in which the type is observed. In this way, the inclusion of species-specific DC types in DC-CL facilitates understanding of the similarities and differences between mouse and human immunology and improved capacity for generating hypotheses about the human immune response from the interpretation of the results of mouse experiments. In this way DC-CL also fosters the advance of translational medicine.

To define cell types on the basis of species of origin, or to include assertions that hold for the type in one species but not another, we recommend the creation of species-specific extensions rather than the inclusion of such types in CL or DC-CL. This approach allows for the representation of detailed, species-specific information without using multiple modes of classification (structure and species of origin) or including conflicting assertions in the core ontology. The approach of more specific extensions of a core template ontology has been used successfully in the creation of species-specific anatomy ontologies as extensions of the Common Anatomy Reference Ontology (CARO) [38] and in the creation of ontologies of specific infectious diseases as extensions of the core Infectious Disease Ontology (IDO) [39].

The use of specific combinations of surface proteins to define DC subtypes has advantages both for the creation of DC-CL and for its application to the analysis of cellular data. A primary means by which experimentalists distinguish cell types is by distinguishing patterns of protein expression using flow cytometry. Defining DC subtypes in terms of flow markers allows easy incorporation into DC-CL of new discoveries about DCs deriving from experiments involving flow cytometry to isolate or analyze cell populations. Similarly, defining DC subtypes in terms of flow markers optimizes DC-CL for the annotation, analysis, and integration of flow cytometry data and of data deriving from experiments in which fluorescence-activated cell sorting is used as a source of cells. Just as the Gene Ontology has been shown to offer significant benefits for the computational analysis of high-throughput data in the study of gene expression using hybridization microarrays [8], we anticipate similar benefits from the use of an ontology of cell types to support analysis of high-throughput, multidimensional flow data.

The relations *has_high_amount *and *has_low_amount *defined in terms of the geometric mean are used in the definition of cell types and are not meant to replace more complicated statistical methods for the analysis of flow cytometry data, such as is described in [40], or other cellular data. Such statistical methods can be applied to the analysis of individual flow data sets, while ontology definitions need to hold universally, across different experimental designs, protocols, and equipment and across differences in the resulting distributions of fluorescence intensities for reference cells. Indeed, the ontology definitions should hold across different assays for surface protein expression, and should not be tied directly to flow cytometry. We have therefore taken a relatively simple approach to the formulation of cell definitions that hold universally and that are supported by our current understanding of DC biology. It is our hope, however, that our work, taken together with [40], will encourage the use of more objective criteria in the analysis of flow cytometry data and in the description and analysis of cell types in general.

The classification of DCs is still an area of active research, thus DC-CL will continue to undergo revisions to keep current with new research results and new technologies for the characterization of cell types [28]. Because ontologies are based on an open world assumption, in contrast to relational databases, they are easily extended to include new subcategories. In addition, the formulation of DC-CL definitions as logically conjoined statements of the from X*R*Y makes it easy to add or remove surface proteins from the definition of any cell type and to use a reasoner to assess consequences of the revision on the DC-CL hierarchy. Thus, newly discovered surface markers can be easily incorporated into the ontology. Furthermore, the system we have outlined is readily applicable to subcellular localizations other than the cell surface and to other cellular components such as mRNA molecules or cytoplasmic granules. In addition to defining more localization-specific relations like *has_plasma_membrane_part*, the general RO relations *has_part *and *lacks *can be used. For all of these relations, too, cellular components other than proteins and protein complexes can be used as arguments. Morphological characteristics such as size and shape can also be used to define cell types using the *has_quality *relation to link to the relevant qualities in PATO, the ontology of phenotypic qualities [41]. In this way, the DC-CL framework lends itself quite readily to the incorporation of new information as knowledge of DC biology increases.

We have built our representations of cell types in DC-CL by relating terms in the domains covered by the OBO Foundry ontologies using relations from the Foundry's relation ontology (RO) and creating new relations as needed. The OBO Foundry [6] was created in 2006 by a group of developers of OBO ontologies on the basis of an evolving set of principles designed to foster the pursuit of best practice in ontology development [13]. Its ontologies are designed to represent in an interoperable fashion the biomedical reality from which data are sampled. Their development within the framework of a common top-level ontology (Basic Formal Ontology, BFO, [42]) and consistent employment of a common set of relations allows Foundry ontologies to be used together as modules of a larger system.

There are many benefits to building DC-CL from OBO Foundry ontologies. In addition to the formalism underlying Foundry ontologies ensuring their support for sophisticated computation both within and between ontologies, building from Foundry ontologies means extensive use of existing ontology resources, both eliminating redundant effort and providing a significant head-start to ontology development. By building on OBO Foundry ontologies, DC-CL is automatically interoperable with other ontologies that also build from Foundry ontologies and with the large information resources, such as UniProt, that use Foundry ontologies for their annotations, representing a wide base of existing annotations. Finally, as OBO Foundry ontologies, and in particular GO, are widely used, use of Foundry ontologies in constructing DC-CL improves the chances that DC-CL will be accepted by the biological ontology and database communities.

DC-CL will serve as a valuable information resource not only providing centralized access to existing information about DCs, but also providing standardized representations that allow algorithmic processing for data analysis and the testing of hypotheses. The consistent use of formally defined relations means that reasoners, such as those included in ontology editing software like OBO-Edit and Protégé, can be reliably applied to DC-CL. In addition, representing information in DC-CL in the from of X*R*Y statements, rather than in natural language definitions, means that DC-CL can be easily parsed, facilitating the implementation of custom algorithms for querying DC-CL or analyzing data annotated in its terms. For example, DC-CL can be queried for the list of proteins expressed by a certain cell type, for the list of cells that express a particular combination of proteins, or for the types of cells that participate in a particular process or have a particular function. We are currently working to integrate DC-CL into software designed for the analysis of flow cytometry data and to assess the ways in which the use of DC-CL can enhance flow data analysis.

## Conclusion

DC-CL provides a prospectively highly valuable resource for the study of DCs. It further offers a generalizable method for the ontological representation of cells that offers significant benefits in the form of increasing the amount and accuracy of information contained in the ontology, enhancing support of the ontology for computation, and providing a much needed resource to support analysis of high-throughput, multidimensional flow data. Thus, we propose the method used to create DC-CL as a strategy for the representation of all cells of hematopoietic lineage.

## Abbreviations

CL: Cell Ontology; DC-CL: Ontology of Dendritic Cell Types; DC: dendritic cells; RO: Relation Ontology; PRO: Protein Ontology; GO CC: Gene Ontology Cellular Component Ontology; CD8α^+^: possession of CD8α molecules on the cell surface (similar notation is used for other molecules); CD8α^-^: absence of CD8α molecules on the cell surface (similar notation is used for other molecules); CD11c^high^: high levels of CD11c molecules on the cell surface (similar notation is used for other molecules); CD11c^low^: low levels of CD11c molecules on the cell surface (similar notation is used for other molecules); TLR: toll-like receptor; CARO: Common Anatomy Reference Ontology; IDO: Infectious Disease Ontology; BFO: Basic Formal Ontology; DC-KB: Dendritic Cell Knowledge Base.

## Authors' contributions

AMM created both the Protégé and OBO-Edit versions of DC-CL, wrote all cell type definitions, participated in the formalization of the defined relations, and drafted the manuscript together with BS and LGC. CNA curated into the Protein Ontology all proteins referred to in DC-CL assertions and revised the manuscript. ADD and RHS contributed to the development of the conceptual framework for the revised Cell Ontology design and revised the manuscript. AEL participated in critical evaluation of the Cell Ontology, which formed the basis for developing the representation of cell types described in the manuscript. CJM participated in the formalization of the defined relations and revised the manuscript. BS participated in critical evaluation of the Cell Ontology, in developing the conceptual framework for the revised Cell Ontology design, in formalization of the defined relations, and in drafting the manuscript. LGC performed the critical evaluation of the Cell Ontology, conceived the described representation of cell types, formalized the defined relations together with BS, and drafted the manuscript together with BS and AMM. All authors read and approved the final manuscript.
